# CLARK TIBBITTS AWARD AND HIRAM J. FRIEDSAM MENTORSHIP AWARD LECTURES

**DOI:** 10.1093/geroni/igz038.677

**Published:** 2019-11-08

**Authors:** Cynthia Hancock

**Affiliations:** University of North Carolina, Charlotte, Charlotte, North Carolina, United States

## Abstract

AGHE’s Clark Tibbits Award was established in 1980 and named for an architect of the field of gerontological education. The award is given each year to an individual or organization that has made an outstanding contribution to the advancement of gerontology and geriatrics education. The Clark Tibbits Award lecture will feature an address by the 2019 award recipient, David Burdick, PhD, of Stockton University. Hiram J. Friedsam was the professor, co-founder, and director of the Center for Studies in Aging and dean of the School of Community Service at the University of North Texas. Dr. Friedsam was an outstanding teacher, researcher, colleague, and mentor to students, faculty, and administrators, as well as a past president of AGHE. The purpose of this award is to recognize those who emulate Dr. Friedsam’s excellence in mentorship. The Hiram J. Friedsam Award lecture will feature an address by the 2019 award recipient, Bradley Fisher, PhD, of Missouri State University.

